# Multiple serial correlations in global air temperature anomaly time series

**DOI:** 10.1371/journal.pone.0306694

**Published:** 2024-07-09

**Authors:** Meng Gao, Xiaoyu Fang, Ruijun Ge, You-ping Fan, Yueqi Wang

**Affiliations:** 1 School of Mathematics and Information Sciences, Yantai University, Yantai, China; 2 Yantai Institute of Coastal Zone Research, Chinese Academy of Sciences, Yantai, China; URV: Universitat Rovira i Virgili, SPAIN

## Abstract

Serial correlations within temperature time series serve as indicators of the temporal consistency of climate events. This study delves into the serial correlations embedded in global surface air temperature (SAT) data. Initially, we preprocess the SAT time series to eradicate seasonal patterns and linear trends, resulting in the SAT anomaly time series, which encapsulates the inherent variability of Earth’s climate system. Employing diverse statistical techniques, we identify three distinct types of serial correlations: short-term, long-term, and nonlinear. To identify short-term correlations, we utilize the first-order autoregressive model, AR(1), revealing a global pattern that can be partially attributed to atmospheric Rossby waves in extratropical regions and the Eastern Pacific warm pool. For long-term correlations, we adopt the standard detrended fluctuation analysis, finding that the global pattern aligns with long-term climate variability, such as the El Niño-Southern Oscillation (ENSO) over the Eastern Pacific. Furthermore, we apply the horizontal visibility graph (HVG) algorithm to transform the SAT anomaly time series into complex networks. The topological parameters of these networks aptly capture the long-term correlations present in the data. Additionally, we introduce a novel topological parameter, Δ*σ*, to detect nonlinear correlations. The statistical significance of this parameter is rigorously tested using the Monte Carlo method, simulating fractional Brownian motion and fractional Gaussian noise processes with a predefined DFA exponent to estimate confidence intervals. In conclusion, serial correlations are universal in global SAT time series and the presence of these serial correlations should be considered carefully in climate sciences.

## Introduction

Air temperature serves as a cornerstone measurement in characterizing Earth’s climate. Since the onset of the industrial revolution, the global average air temperature has been steadily increasing, and the scientific consensus on global warming is unequivocal, as evidenced in studies by Hansen et al. [1] and Hartmann et al. [2]. Air temperature time series are quintessential examples of climate data, where discernible trends mirror global warming, while periodic variations reflect seasonality or broader climate variability, as discussed in works by Gao et al. [3] and Fanzke et al. [4]. Once the linear trend and seasonal patterns are removed, the remaining component of the air temperature time series is referred to as temperature anomalies. These anomalies allow for the identification of heat and cold waves, crucial indicators in understanding global climate change, particularly the evolution of climate extremes, as highlighted by Wang et al. and Gao et al. [5, 6]. A thorough analysis of these temperature anomaly time series is pivotal in gaining a comprehensive understanding of global climate dynamics [7].

Serial correlation is a pivotal statistical metric that quantifies the correlation between a variable’s current state and its historical states. The presence of serial correlation signifies that the time series exhibits a deviation from randomness, demonstrating a form of temporal persistence [8]. When considering climate change, this temporal persistence of extreme weather events holds immense significance for the resilience of natural ecosystems and human society. A recent study by Li and Thompson revealed widespread alterations in temporal persistence under projected climate change scenarios for the 21st century [9]. Serial correlation is a common occurrence in empirical time series data in climatology and environmental sciences [10–13]. Distinguishing these correlations across different scales is imperative for unraveling the intricate dynamics of climate time series and the underlying mechanisms that drive them [13–16]. Short term correlation (STC) in climate time series refers to the relationship between climate variables over a relatively brief period, such as a few days, which is typically influenced by weather patterns, atmospheric oscillations [10, 17]. Long-term correlation (LTC) in climate science refers to the relationship between climate variables over extended periods, often months, years, decades and even centuries. This type of correlation is generally driven by larger-scale processes such as climate change, natural cycles, and anthropogenic emissions [10, 18–20]. Nonlinear correlation (NC) reflects the nonlinearity and complexity of stochastic climate time series [21]. Time series of air temperature anomalies reflect the complex dynamics of climate system on different scales; thus, it is actually a mixture of white noise (uncorrelated), STC, LTC and NC [22–24]. In this study, we will investigate the serial correlation structure of daily time series of global surface air temperature (SAT) anomalies.

Autocorrelation, commonly known as lagged correlation, serves as a potent tool for analyzing serial correlation. Traditional time series analysis methods relied heavily on the linearity assumption of the underlying processes, where autocorrelation functions (ACFs) typically exhibit exponential decay. For white noise, the ACF, the ACF, *c*(*τ*), is 0 for any time lag *τ*. However, many natural time series exhibit an exponentially decaying ACF beyond a specific time lag *τ* > *τ*_0_, indicating the presence of STC. Conversely, when ACFs follow a power-law decay function, *c*(*τ*) ∼ *τ*^−*γ*^, the original time series is considered to exhibit LTC. LTC is also referred to as long memory, long-term persistence, Hurst phenomenon, or long-range dependence in various literatures [13, 15, 25]. Through ACFs, one can estimate the duration of temporal persistence by setting a threshold correlation value [9, 26]. Apart from ACFs, detrended fluctuation analysis (DFA) is a well-established method for detecting LTC. The scaling exponent *α* quantifies the strength of linear correlations within the time series. Values of *α* < 0.5 indicate anti-correlation, while *α* = 0.5 suggests an uncorrelated time series. An *α* greater than 0.5 but less than or equal to 1.0 signifies persistent long-term correlations. When *α* > 1, correlations are present, but the ACF no longer follows a power-law form. A special case of *α* = 1.5 represents brown noise, which is essentially the integration of white noises [27]. In this study, we aim to detect both STC and LTC in global surface air temperature (SAT) time series using these statistical methods.

In the real world, various time series exhibit diverse scaling behaviors for fluctuations of different magnitudes, leading to the concept of multifractality [6, 27–31]. To detect this multifractality, numerous techniques have been introduced, with the multifractal detrended fluctuation analysis (MFDFA) emerging as a particularly successful approach [32]. MFDFA builds upon the classical DFA method by identifying the scaling of q-order moments based on the time series length. In MFDFA, the width of the multifractal spectrum serves as a conventional measure to quantify the degree of multifractality (or nonlinearity) within the time series [32]. Furthermore, the nonlinearity of the original time series can be assessed by applying DFA or MFDFA to the decomposed series of magnitude and sign derived from the original time series [24, 33–35]. However, some studies have indicated that the presence of correlation in the magnitude series alone is not a definitive indicator of nonlinearity in the original time series [21]. Additionally, the successful application of DFA and MFDFA is influenced by factors such as the polynomial order, the scaling region, and the probability distribution function (PDF) of the series [21]. In this study, nonlinearity is treated as a proxy for multifractality and estimated using a novel method based on complex network analysis.

Over the past two decades, complex network approaches have gained significant popularity in quantifying structural properties of time series. A diverse array of algorithms has been developed to transform a single time series into a complex network, including but not limited to those proposed by Marwan et al. [36], Lacasa et al. [37], Barreiro et al. [12], and Tsiotas et al. [38]. The visibility graph (VG) algorithm introduced by Lacasa et al. [37] maps time series into complex networks, effectively preserving the serial properties of the original data. This allows for the correlation information within the time series to be inferred from the topological properties of the resulting network [37, 39, 40]. A simplified version of the VG, known as the horizontal visibility graph (HVG), has demonstrated higher efficiency in practical applications [41–47]. Manshour et al. [48] calculated three topological parameters (TPs) of HVG-mapped complex networks and observed monotonic relationships between these TPs and long-term correlations (LTC), suggesting the potential to infer LTC strength in idealized fractional time series from complex network analysis. However, Huang et al. [24] demonstrated that in practical applications, the inference of LTC parameters from complex networks can be influenced by the combined effects of short-term correlations (STC) and nonlinearity (NC). Nevertheless, it has been verified that the HVG method can still be applied to detect NC by eliminating linear correlations and calculating a newly proposed topological parameter, Δ*σ*. This detection method is robust and unaffected by the probability distribution of the time series [21].

The objectives of this study include two aspects: (i) verify the presences of three kinds of serial correlations (STC, LTC and NC) in global SAT anomaly time series; (ii) interpret these spatial patterns of serial correlations from the view of large-scale atmospheric processes or climate variability. In this study, STC and LTC in global SAT anomalies time series will be detected by two traditional methods, i.e. AR(1) and DFA, and the length of temporal persistence is also estimated based on ACFs. Moreover, NC will be detected by calculating the TP (Δ*σ*) of HVG mapped complex networks after eliminating the effects of linear correlations. Specifically, we propose to apply Monte Carlo method to test the statistical significance of Δ*σ* in this study. This paper is organized as follows. The data and statistical methods used in this study are presented in the next section. Results and discussions are shown in the third and fourth sections, respectively. Lastly, the main findings of this study are concluded in the final section. All the abbreviations utilized in this study are listed in S1 Appendix.

## Materials and methods

### Data

**Fig 1:**
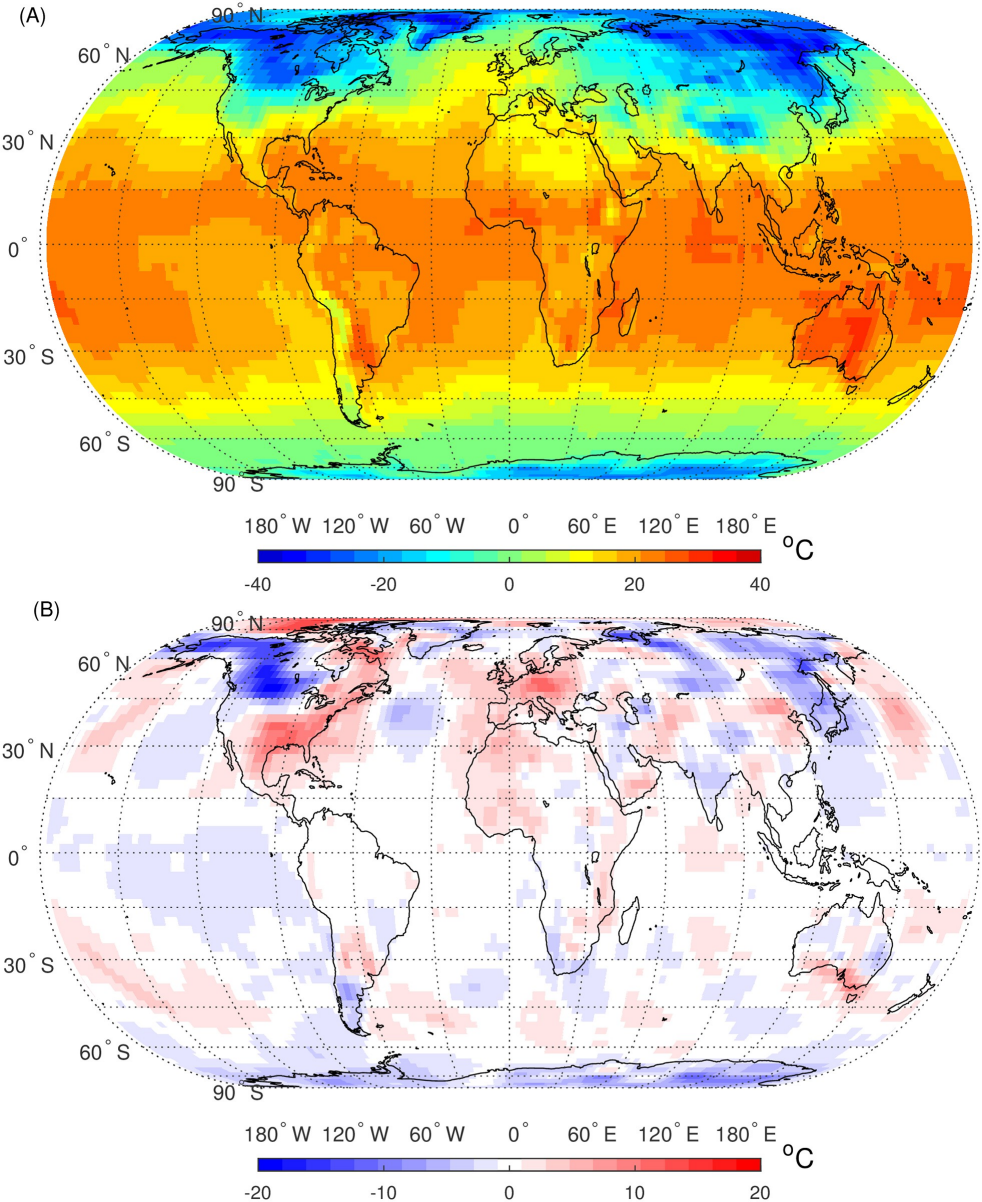
Global maps of SAT (A) and SAT anomalies (B) on Dec. 31, 2021. Shapefiles are downloaded from Natural Earth, http://www.naturalearthdata.com/.

In this study, datasets of global surface air temperature extracted from NCEP/NCAR Reanalysis-1 are analyzed. The datasets can be directly accessed from the following website https://www.weather.gov/ncep/. The time range of the datasets is from 1948 to 2021 lasting for 74 years, and the spatial resolution is 2.5° × 2.5°. The daily time series of SAT anomalies are calculated by subtracting the mean annual cycle and the linear trend sequently from the original SAT time series [3, 4, 24]. Specifically, the mean annual cycle is calculated by averaging the daily temperatures over the entire 74 years of analysis. Then, the linear trends are estimated by the linear regression. Thus, linear correlations (STC and LTC) caused by seasonality and linear trend are eliminated in the SAT anomaly time series. Then, we get 10512 time series in total covering the Earth’s whole surface. The lengths of each time series are 27010, where the data on Feb.29th in all leap years are removed ensuring that the lengths in each year are equal. The length is sufficiently long for DFA and HVG analyses. As an illustration, Fig 1 presents the global SAT and SAT anomalies on the last day of the study period, Dec. 31, 2021.

### Lagged correlation analysis

In this study, STC in SAT anomaly time series is detected by fitting time series to the first-order autoregression model, AR(1):

$$\begin{array}{c} X_{t}=\rho X_{t-1}+\omega_{t} \end{array}$$ (1)

where *X*_*t*_ represents the objective time series, and *X*_*t*_ is the function of *X*_*t*−1_. *ω*_*t*_ is a normally distributed independent error term. The first-order autocorrelation coefficient, *ρ*, measures the correlation between *X*_*t*_ and *X*_*t*−1_, and *ρ* is also known as the lag 1 autocorrelation. A positive *ρ* indicates the evidence of STC in the time series. Moreover, the length of temporal persistence is also evaluated based on the smoothed ACFs using the Savitzky-Golay filter with width 15 days and polynomial order three [26]. The length of temporal persistence is also defined as the dataset length from lag 1 to the lag with a correlation of *r* = 0.6.

### Detrended fluctuation analysis (DFA)

DFA was original proposed as an alternative method enabling correct estimation of the power law scaling (Hurst exponent) in time series analysis in the presence of (extrinsic) nonstationaries, so that the spurious detection of LTC could be avoided [27, 49–52]. More practical applications show that DFA performs better than other heuristic techniques such as the Rescaled Range (R/S) Analysis even for the fractional Brownian noise. The basic idea of DFA is to quantify fluctuations around local trends as

$$\begin{array}{c} F^{2}\left(n\right)=\frac{1}{mn}\sum_{j=0}^{m-1}\sum_{i=(j+1)n}^{(j+1)n}\left(X_{i}-{\tilde{X}}_{i}^{j}\right) \end{array}$$ (2)

where *X*_*i*_ is the objective time series and ${\tilde{X}}_{i}^{j}$ represents a local trend in the time interval [*jn* + 1, (*j* + 1)*n*]. Thus, *n* is the length of the time interval, and *m* is the number of time intervals. If the local trend ${\tilde{X}}_{i}^{j}$ is obtained by fitting a linear function or n-th order polynomial to *X*_*i*_ in the time interval [*jn* + 1, (*j* + 1)*n*], and the corresponding method is denoted as DFA or DFAn, respectively. If there is a power law scaling, the scaling exponent *α* could be approximated by the slope of linear regression model *log*_10_(*F*^2^(*n*)) vs *log*_10_(*n*). In this study, the original SAT time series have been preprocessed, then the nonstationaries caused by trend and seasonality that limits the effectiveness of DFA could be been avoided.

### Horizontal visibility graph (HVG) algorithm

HVG is a simpler version of VG approach with the advantage of providing analytical expressions for fully random time series [39]. In VG and HVG, each observation *X*_*t*_ in the time series is considered as a “node”, and the definition of VG for time series comes from the concept of visibility between nodes. Any two nodes are considered to be connected if they can “see” each other implying that there is a straight line connecting them without being interrupted by other intermediate nodes. For example, two nodes *X*_*a*_ and *X*_*b*_ are connected if another other node *X*_*c*_ between them satisfy the following condition:

$$\begin{array}{c} X_{c}\leq X_{b}+\left(X_{a}-X_{b}\right)\frac{t_{a}-t_{c}}{t_{b}-t_{a}} \end{array}$$ (3)

where *t*_*a*_, *t*_*b*_, and *t*_*c*_ are the occurring time of *X*_*a*_, *X*_*b*_, and *X*_*c*_, respectively. When the above condition is relaxed to *X*_*c*_ ≤ *min*{*X*_*a*_, *X*_*b*_} and nd the straight line is restricted as a horizontal line, the VG algorithm becomes the HVG algorithm. In other words, horizontal visibility for two nodes occurs if there is no other node greater in magnitude between them; therefore, they are connected by an “edge”. A complex network constructed with this criterion is called HVG and usually denoted as *G*(*N*, *E*). The degree of a node in a complex network is the number of edges connected to it, and we denote *k*_*t*_ as the degree of node *X*_*t*_. Then the degree distribution *p*(*k*) will be fitted to an exponential function:

$$\begin{array}{c} p\left(k\right)∼e^{-\lambda_{c}k} \end{array}$$ (4)

and λ_*c*_ will be estimated in the semi-logarithmic plots of log(*p*(*k*)) vs *k*. In this study, we also calculated another TP of complex networks (the Spearman correlation between *X*_*t*_ and *k*_*t*_) to explore the possibility of inferring LTC from HVG mapped complex networks for SAT anomaly time series. The range of the Spearman correlation is also within [−1, 1], and more details about the definitions and calculations could be found in refs [24, 48].

### Detection of nonlinear correlation (NC)

NC will be measured by using a newly proposed TP of HVG mapped complex network [21]. A new series (*x*_*t*_) of increment magnitude is firstly extracted from the original time series *X*_*t*_ based on the following equation:

$$\begin{array}{c} x_{t}=\left|X_{t+1}-X_{t}\right| \end{array}$$ (5)

The nonlinear correlations in the magnitude series *x*_*t*_ will be eliminated by a phase randomized surrogate procedure. Manshour [21] showed that NC could be measured by the following measure:

$$\begin{array}{c} \Delta \sigma =\frac{|\sigma_{k}^{x}-\sigma_{k}^{x^{RPS}}|}{\sigma_{k}^{x}} \end{array}$$ (6)

where $\sigma_{k}^{x}$ and $\sigma_{k}^{x^{RPS}}$ are the standard deviations of degree distribution of HVG networks for the increment magnitude time series *x*_*t*_ and its phase randomized counterpart. The corresponding relationship between TP and NC has already been verified in two previous literatures [35, 53]. Δ*σ* = 0 implies there is no NC in the original time series, while a positive value of Δ*σ* indicate the presence of NC.

**Fig 2:**
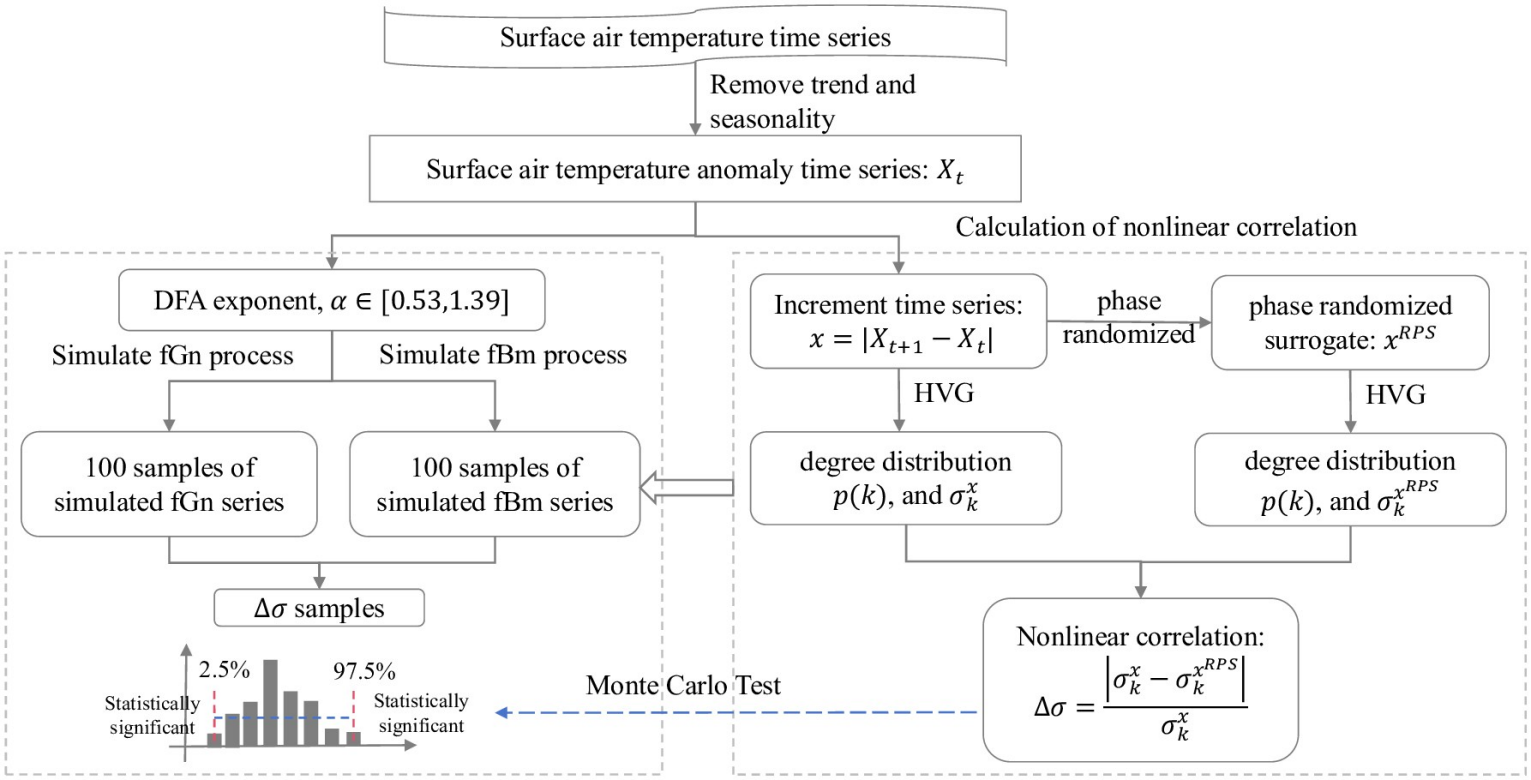
The flow diagram of calculating the topological parameter of HVG mapped complex networks, Δ*σ* and the associated Monte Carlo test.

The statistical significance of nonlinear correlation Δ*σ* is tested by Monte Carlo method. For a given value of DFA exponent *α*, fractional Brownian motion (fBm) and fractional Gaussian noise (fGn) processes are simulated independently, and the length of the generated time series is equals to that of the SAT time series. The fBm process is defined as a stochastic integral with respect to ordinary Brownian motion, while the incremental process of fBm is a stationary discrete-time process called fGn [54]. Both fGn and fBm series have Gaussian PDF and are thought to be linearly correlated without nonlinear correlation [21]. We simulate 100 fGn and fBm series respectively, and estimate the values of Δ*σ*. If the value of Δ*σ* for the empirical time series is lower than 2.5% percentile or larger than 97.5% percentiles of Δ*σ* samples of simulated time series, it is thought to be statistically significant. The flowchart of the calculation of Δ*σ* as well as the associated Monte Carlo tests is presented in Fig 2. The statistical analyses of the global SAT time series as well as the Monte Carlo tests are implemented within the statistical computing environment R [55, 56].

## Results

**Fig 3:**
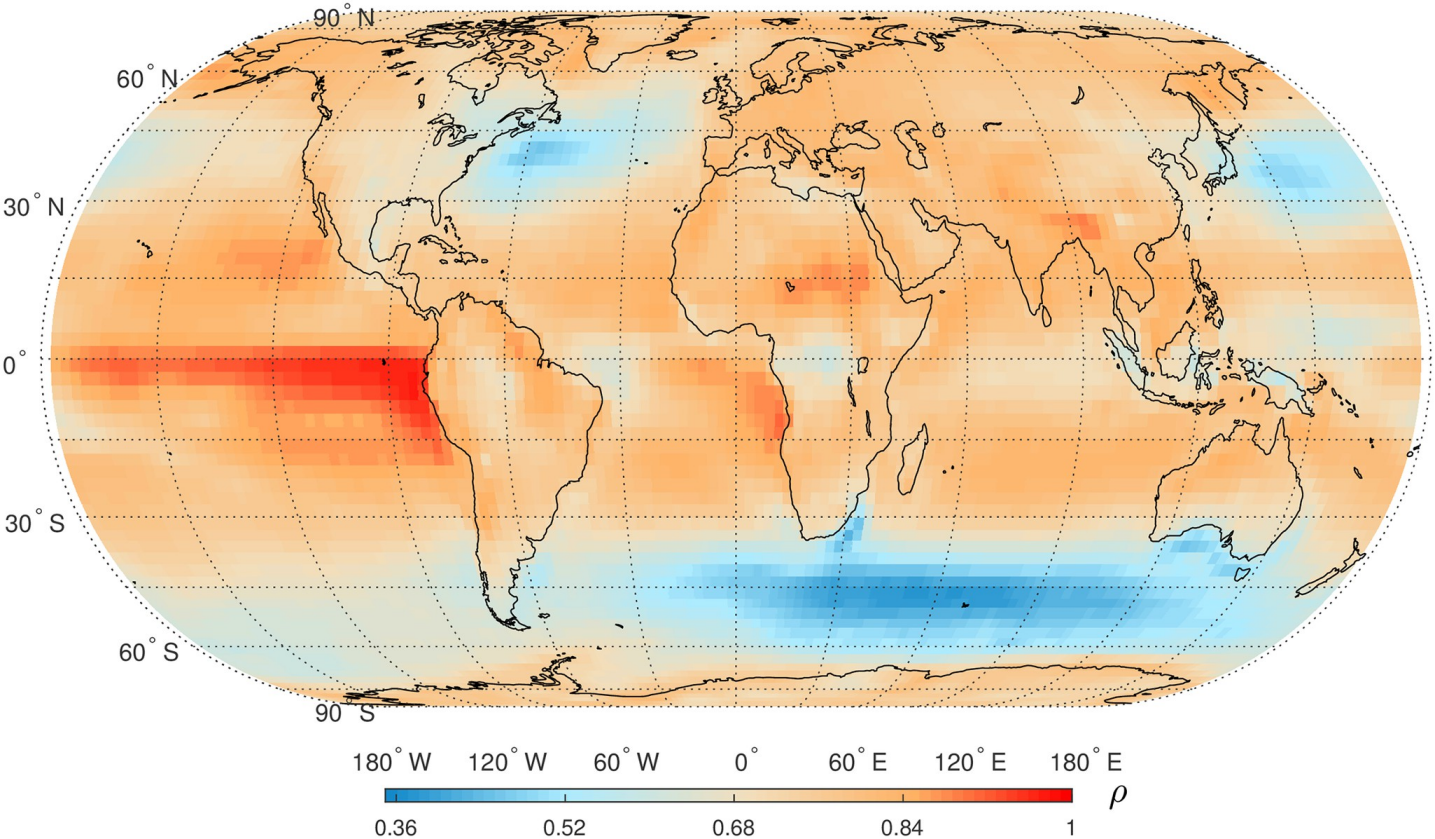
Global map of first-order autocorrelation coefficient, *ρ*, in AR(1) model of daily SAT anomaly time series in the period 1948–2021. Shapefiles are downloaded from Natural Earth, http://www.naturalearthdata.com/.

**Fig 4:**
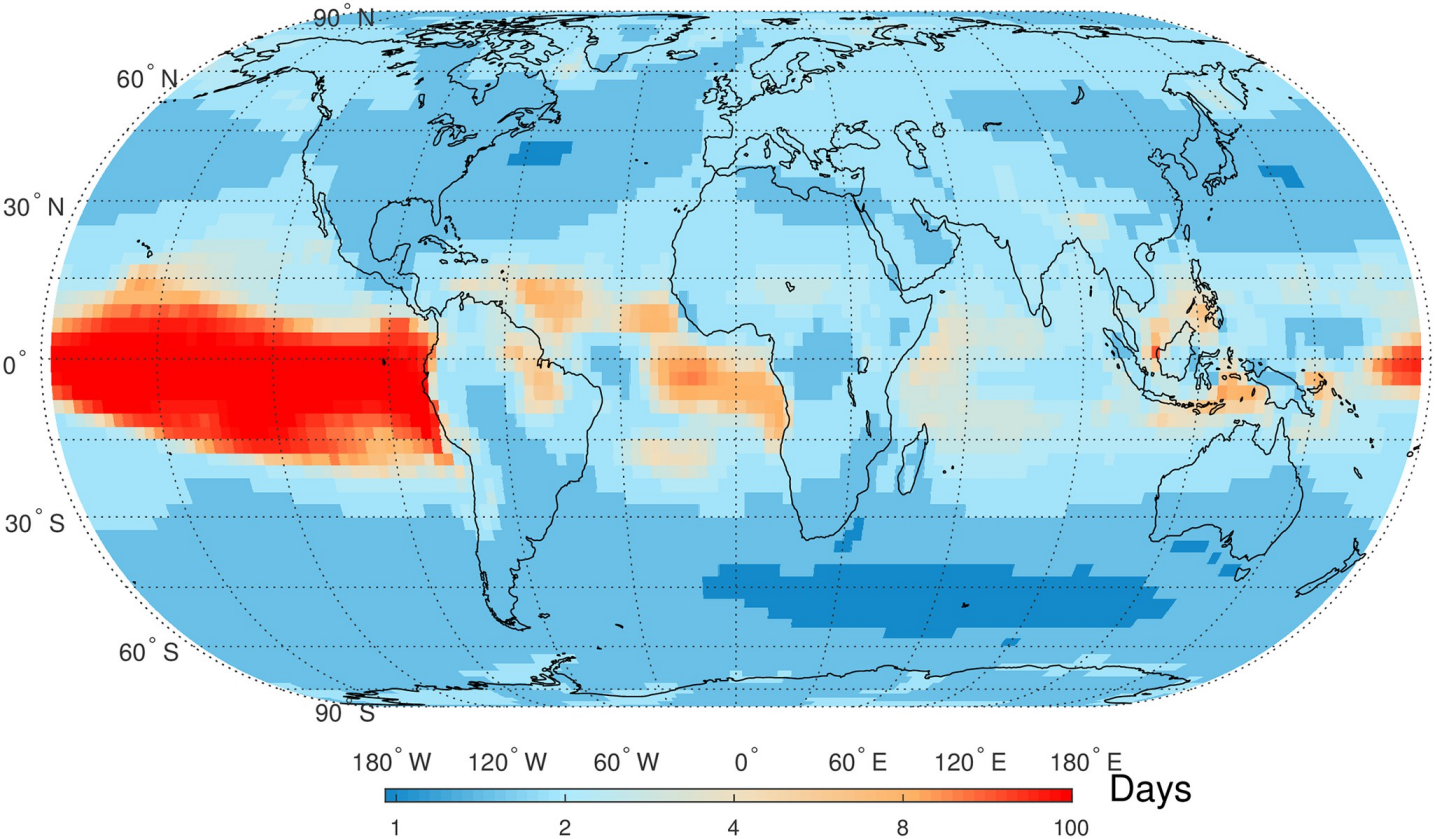
Global map of length of temporal persistence estimated based on the ACFs of daily SAT anomaly time series. Shapefiles are downloaded from Natural Earth, http://www.naturalearthdata.com/.

The detection of STC in global SAT anomaly time series is achieved by estimating the parameter *ρ* in autoregressive models AR(1). Fig 3 depicts the global distribution of the values of *ρ*, where the minimum value is about 0.36 and the maximum value is close to 1. This phenomenon underscores the universal presence of STC in the SAT anomaly time series. Firstly, the *ρ* values over eastern Pacific in tropics are higher than 0.8 indicating the evidence of STC. In tropical and subtropical regions excepts rainforests in African continent and Indonesian Islands, the values of *ρ* are basically larger than 0.6. Moreover, the *ρ* values over South Indian Ocean (35°S-60°S, 30°W-150°E), Northwest Pacific (30°N-45°N, 130°E-175°E), and Northwest Atlantic (35°N-45°N, 40°W-80°W) are lower than those in other areas. Additionally, Fig 4 presents the lengths of persistence of smoothed autocorrelation functions (ACFs) decaying to 0.6. These lengths provide further insights into the strength and duration of STC across different regions. Strong persistence is also located at eastern Pacific in tropics that is consistent with the distribution of *ρ*. The areas with weaker persistence are also consistent with those with lower *ρ* values presented in Fig 3.

**Fig 5:**
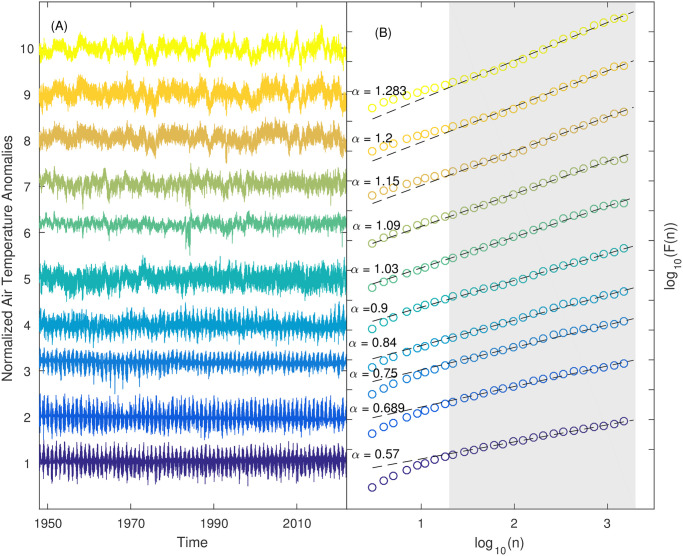
Ten time series of SAT anomalies (A) and their fluctuation functions (B). The time series have been normalized (subtracting the average and dividing by the standard deviation) and placed in one plot, and the DFA exponent *α* is estimated in the double-logarithmic plot. The grey-shaded area displays the window for which the *α* value has been estimated (15 ≤ *n* ≤ 1500 days).

**Fig 6:**
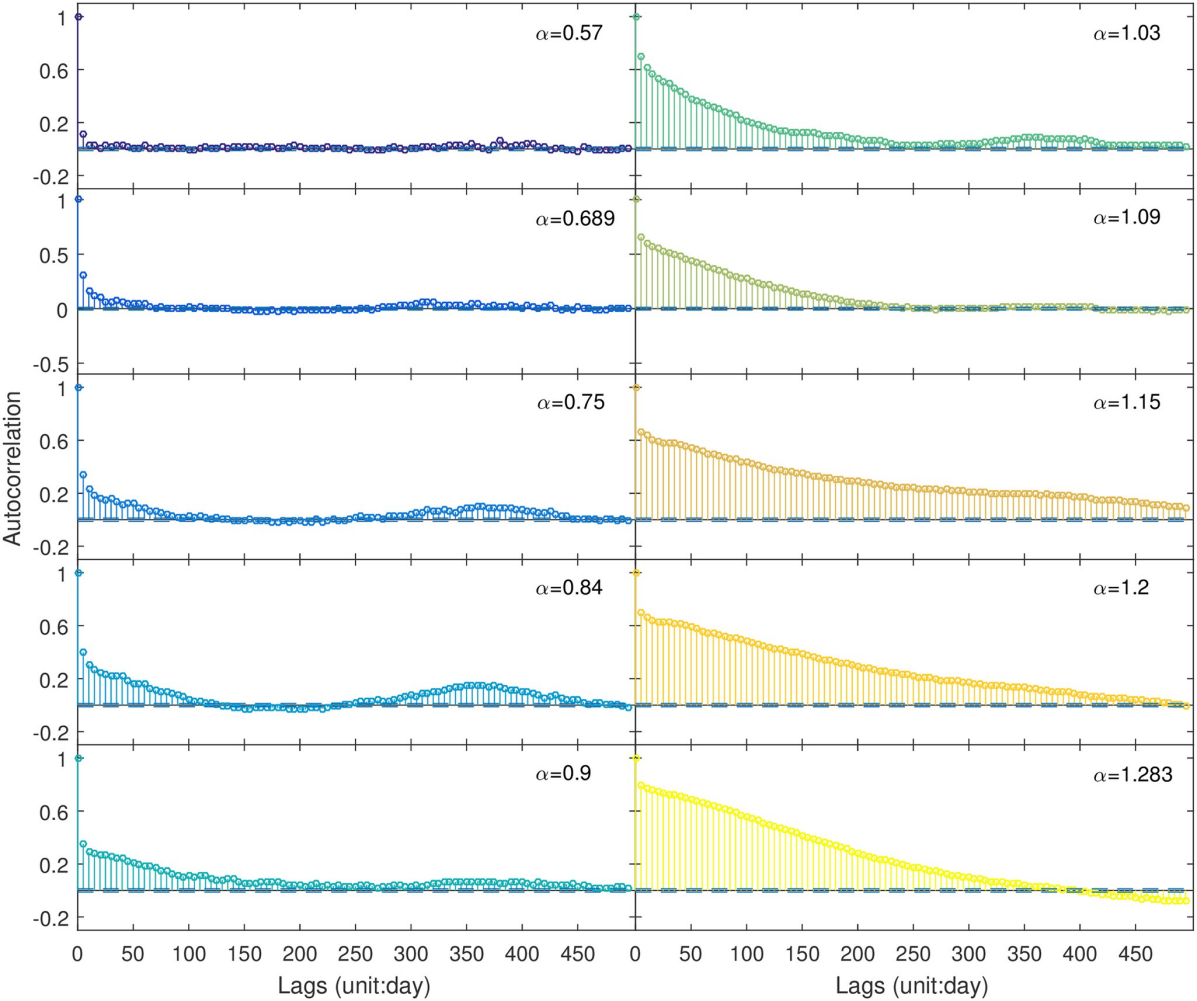
Autocorrelation functions for the ten time series of SAT anomalies presented in Fig 4.

**Fig 7:**
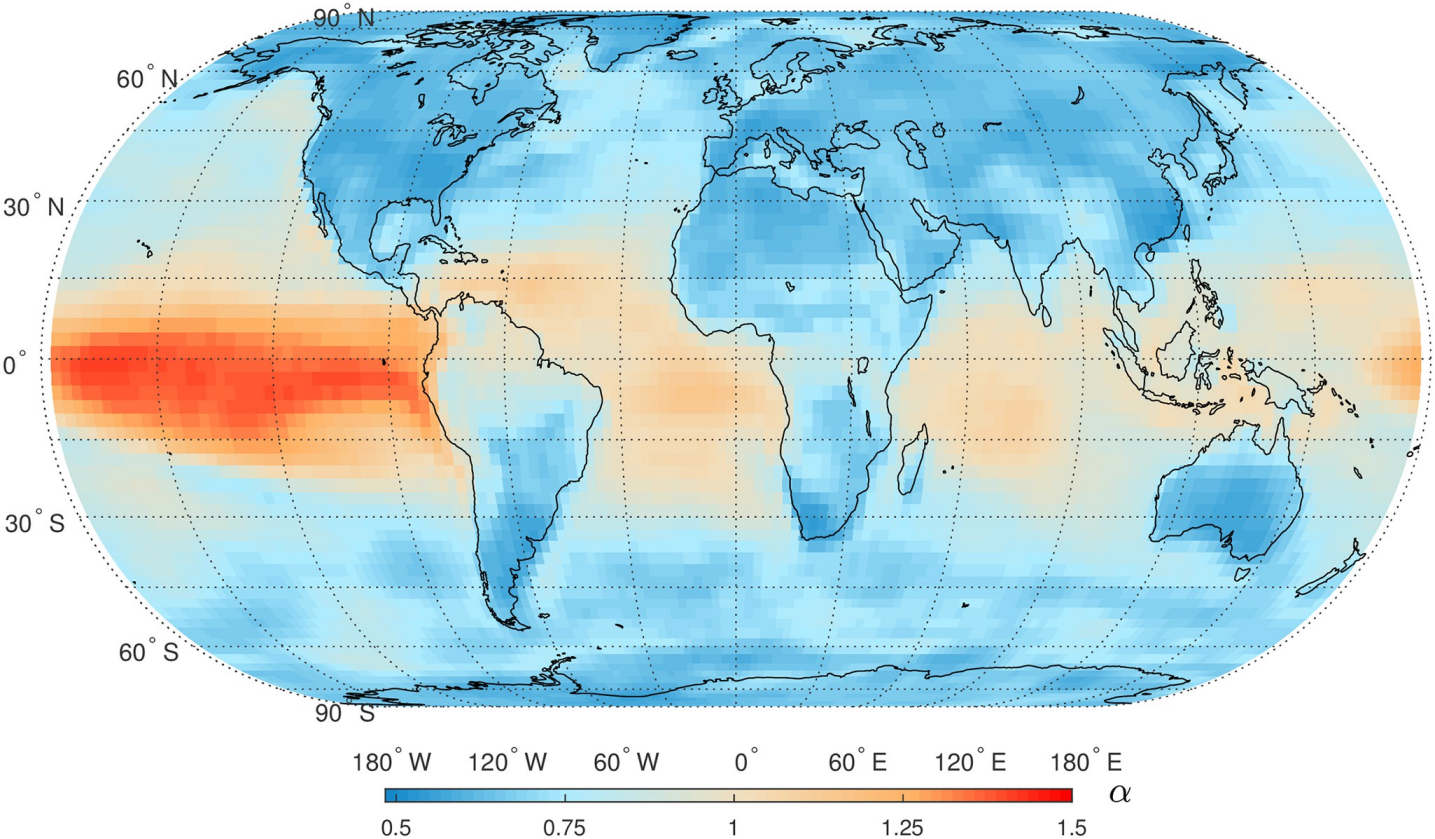
Global map of DFA exponent, *α*, of daily SAT anomaly time series in the period 1948–2021. Shapefiles are downloaded from Natural Earth, http://www.naturalearthdata.com/.

Before applying DFA method to all SAT anomaly time series, we randomly select 10 time series and plot the fluctuation functions to determine a proper scaling region (Fig 5). From Fig 5, we observe a crossover phenomenon around *n* = 14, indicating that the DFA exponent *α* must be extracted from a suitable scaling region to avoid this crossover. Furthermore, we select additional time series and confirmed the crossover phenomenon around *n* = 14 (not illustrated as figures). The presence of this crossover validates the existence of short-term correlations (STC) in the SAT anomaly time series. For the subsequent analysis, we set the scaling region as *n* ∈ [15, 1500]. The estimated values of *α* for these 10 time series range from 0.57 to 1.283, where the “roughness” of these time series decreases accordingly. The ACFs for these ten series are presented in Fig 6, where LTCs are partly reflected from the decaying rules of ACFs. Then, DFA method is applied to all time series, and global patterns of DFA exponent *α* are presented in Fig 7. The minimum value of *α* is about 0.53, and the largest *α* is approximately 1.39. From Fig 7, it is found that larger *α* values are mainly distributed in tropical oceans, especially the eastern Pacific in tropics. This area is consistent with that of higher values of *ρ* representing STC.

**Fig 8:**
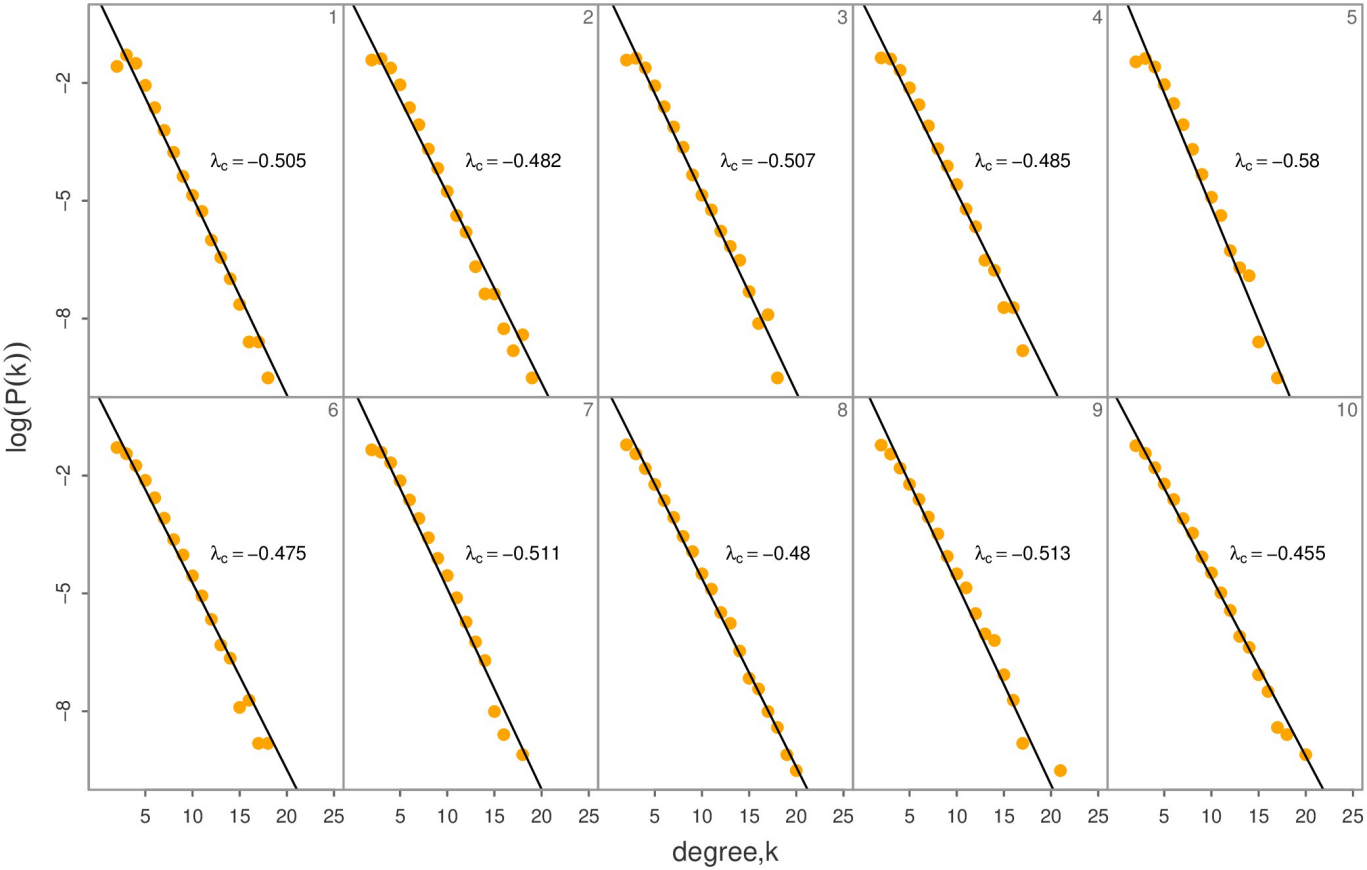
Degree distribution of the ten HVG mapped complex networks for the ten time series in Fig 2. The slopes λ_*c*_ are estimated in the semi- logarithmic plots.

**Fig 9:**
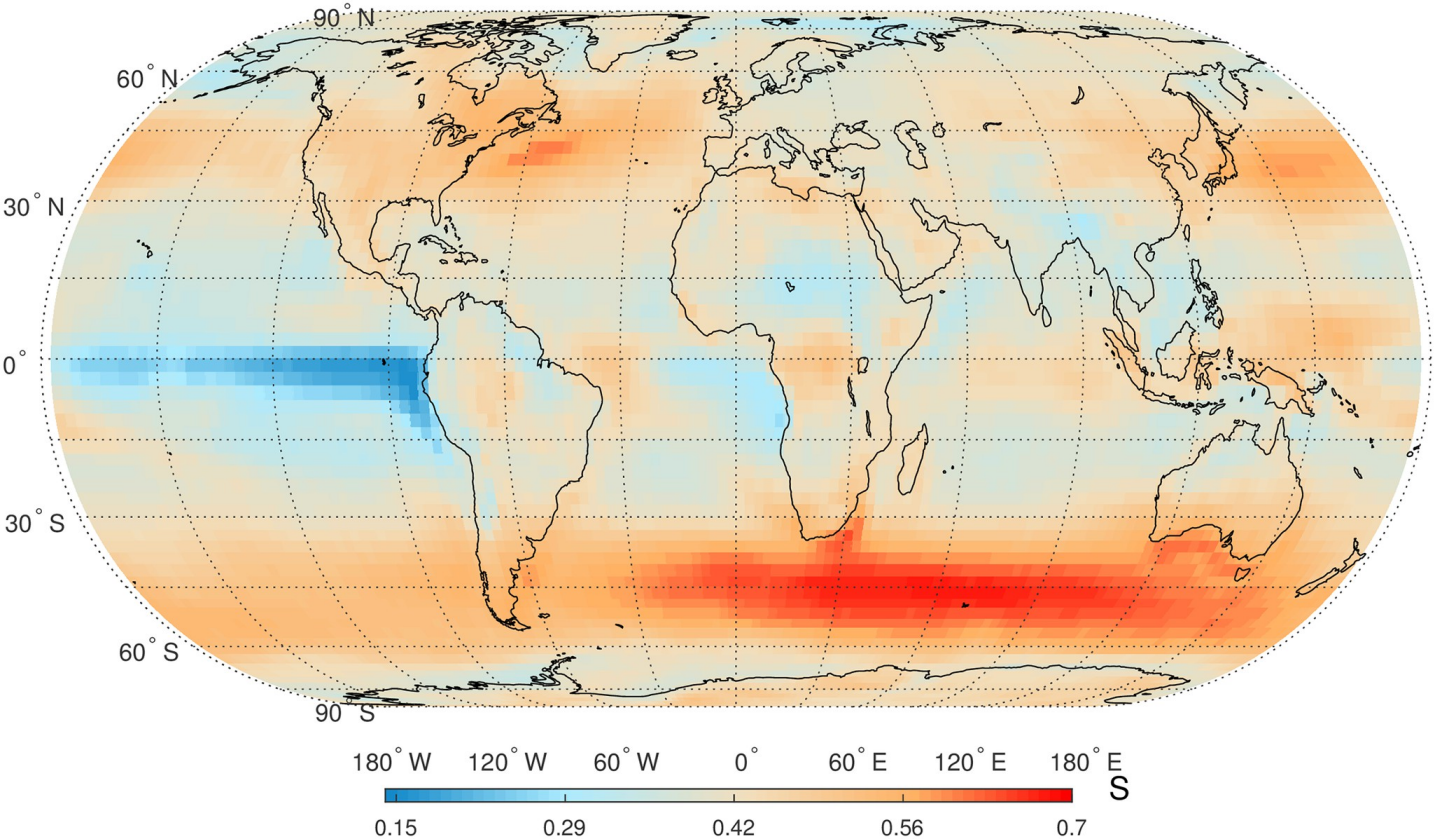
Global map of the topological parameter, Spearman correlation between *X*_*t*_ and *k*_*t*_(*S*), of HVG mapped complex networks for daily SAT anomaly time series. Shapefiles are downloaded from Natural Earth, http://www.naturalearthdata.com/.

Next, all SAT anomaly time series are mapped onto complex networks, and the degrees as well as the degree distributions are calculated, respectively. The degree distribution *p*(*k*) will be firstly fitted to an exponential function for these 10 time series presented in Fig 5, and the linear regression results in the semi-logarithmic plots of log(*p*(*k*)) vs *k* are shown in Fig 8. Although the DFA exponent *α* for these 10 time series increases from 0.57 to 1.283, there is no one-to-one correspondence between *α* and λ_*c*_ for these empirical time series. Our results here are consistent with those found in [24], although such one-to-one correspondence exists for idealized time series with LTC [48]. Therefore, it is not reasonable to detect LTC from λ_*c*_, because empirical time series are usually subject to white noises during measurement and the exponential degree distribution is due to the measurement errors [24]. Then, the Spearman correlations between *X*_*t*_ and *k*_*t*_(*S*), are calculated for the HVG mapped complex networks of global SAT anomaly time series (Fig 9). In ref. [48], there was a monotonically decreasing one-to-one relationship between *α* and *S* for idealized time series with LTC. In this study, we show that this monotonic correspondence does not strictly hold for global SAT anomaly time series except for the Eastern Pacific and Eurasian continent. This result indicates that LTC of empirical time series could be partly inferred from HVG mapped complex networks, and is in line with that found in [24]. Moreover, the spatial pattern *S* is compared to that of *ρ*, and a reverse relationship between *S* and *ρ* has been found (Figs 3 and 9).

**Fig 10:**
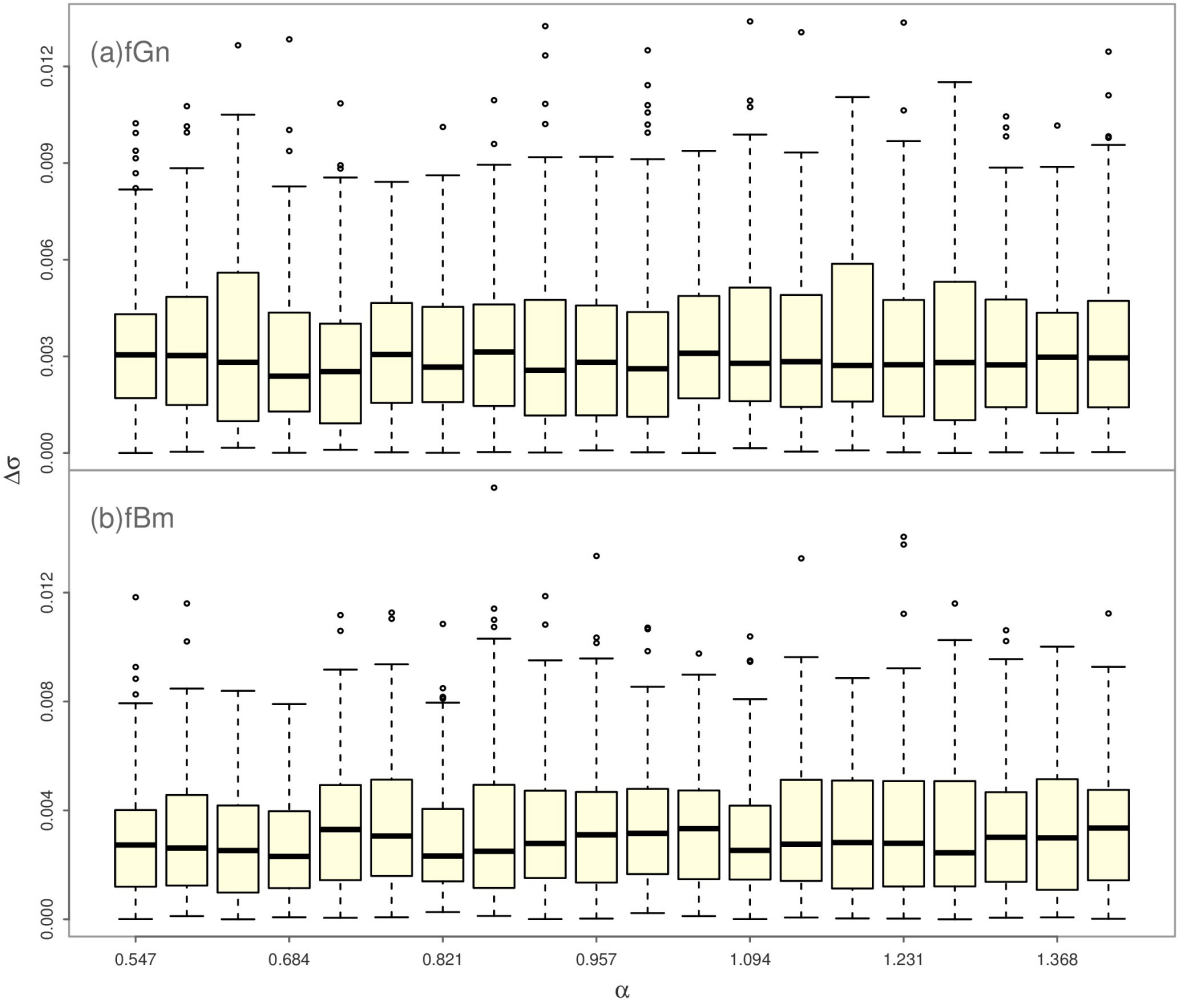
(a) Boxplots of Δ*σ* based on 100 independent simulations of fractional Gaussian noise (fGn) processes with predefined DFA exponent *α*. (b) Boxplots of Δ*σ* based on 100 independent simulations of fractional Brownian motion (fBm) processes with predefined DFA exponent *α*.

**Fig 11:**
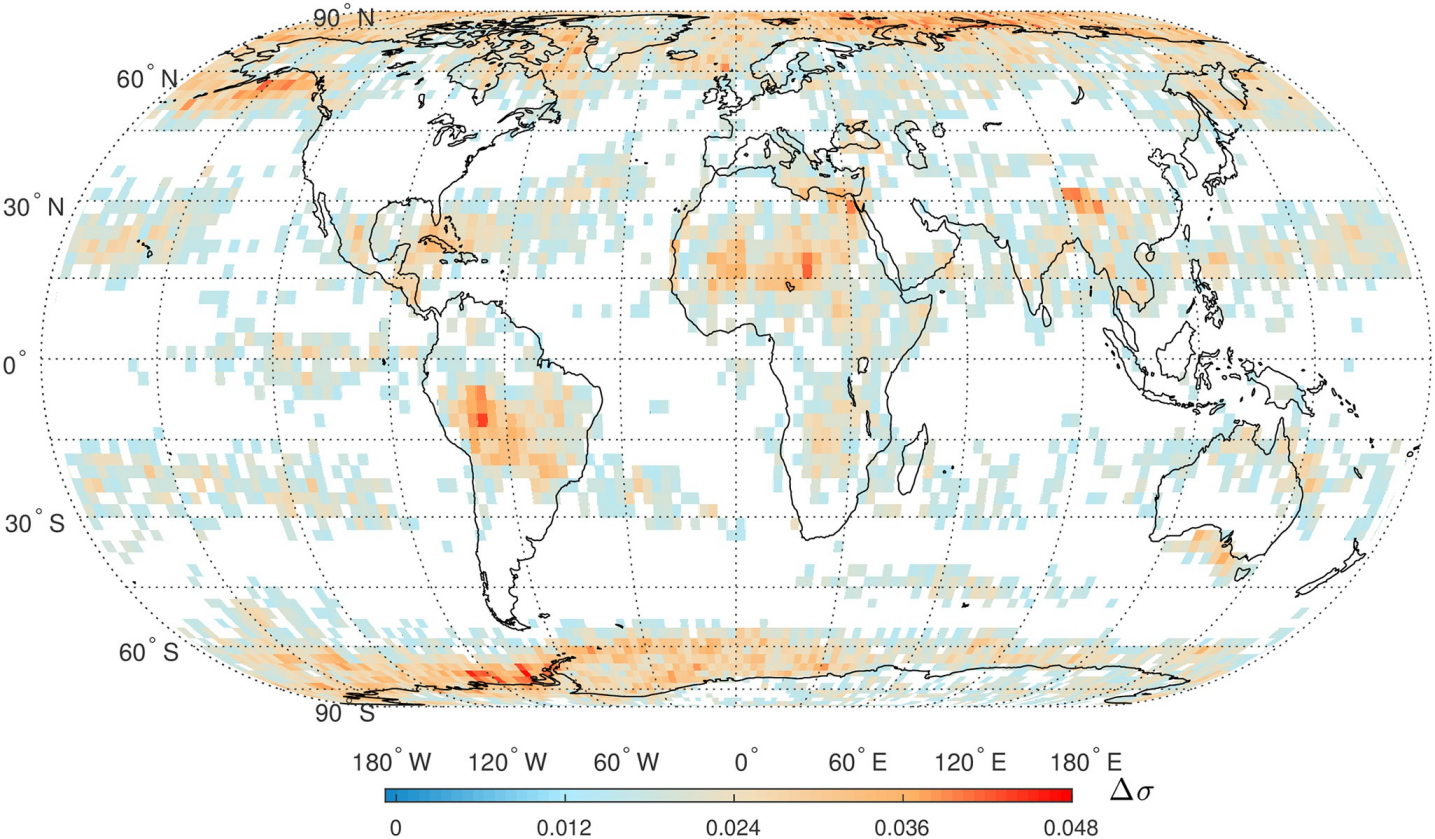
Global map of the topological parameter, Δ*σ*, of HVG mapped complex networks for daily SAT anomaly time series (only statistically significant values are presented). Shapefiles are downloaded from Natural Earth, http://www.naturalearthdata.com/.

Finally, we calculate the newly proposed TP, Δ*σ*, for global SAT anomaly time series. The 95% confidence intervals are firstly constructed by simulating the fBm and fGn processes for a given *α* value, respectively. The range of *α* is in [0.53, 1.39] for global SAT anomaly time series. In this study, we divide [0.53, 1.39] into 20 segments and simulate fBm and fGn processes for a mean value of *α* in these 20 segments. The statistical significance of all Δ*σ* values of global SAT anomaly time series are based on the 95% confidence intervals presented in Fig 10. From Fig 10, it is observed that Δ*σ* is almost independent on the long-term correlation parameter *α* for the fBm and fGn processes. The statistically significant Δ*σ* those are larger than the upper 97.5% bound in Fig 10 are presented in Fig 11. The presence of NC has been identified in a large proportion of SAT anomaly time series, especially in the higher latitude areas, indicating the universality of NC in global SAT anomaly time series. Other regions with higher degree of NC are located near the 15° latitude in the two hemispheres.

## Discussions

Serial correlation is common in all kinds of temperature time series, and the temporal persistence pose a threat to natural ecosystems and human society. In this study, serial correlations in global SAT time series have been investigated. The daily SAT time series extracted from the reanalysis datasets are firstly preprocessed by removing the seasonality and liner trend, so that the linear correlations (STC and LTC) caused by seasonality and linear trend are avoided. Then, the residual time series is referred to as SAT anomaly time series, and the serial correlations reflect the climate variability of Earth’s climate systems. Three kinds of serial correlations have been detected in these SAT anomaly time series by using different statistical methods.

STC captures the dependency of random variables on their previous values within a short time frame. This dependency can be either positive (i.e., similar values tend to follow each other) or negative (i.e., opposite values tend to follow each other). In this study, STC has been detected in global SAT anomaly time series by fitting a simple first-order auto-regressive model, AR(1). Positive STC values indicate that positive correlations are common for global SAT anomaly time series on daily time scale. The eastern Pacific in tropics has the stronger STC compared to other areas, and this phenomenon might be attributable to the influence of Eastern Pacific Warm Pool (EPWP) on SAT [57]. Net heat flux from ocean to atmosphere have been detected in these three regions, which correspond to west wind drift current, Kuroshio current, and Gulf current, respectively [58, 59]. The latitudes of these three areas are basically consistent with that of atmospheric Rossby waves in the two hemispheres [60]. The combined effect of ocean currents and atmospheric Rossby waves might be responsible to the weak STC in these three areas. In the tropical rainforest regions, the *ρ* values are also lower indicting a weak STC in SAT anomaly time series. This finding can be partly explained by the complex land-atmosphere interactions in rainforest regions [61]. Unsurprisingly, the lengths of temporal persistence are largest over EPWP due to the long term impact of El Niño–Southern Oscillation (ENSO), and the temporal persistence in mid-latitude areas is relatively shorter.

Climate variables naturally exhibit variability of warmth and coolness, wetness and dryness over months, years and even decades. LTC has been widely detected in climate sciences, i.e. the time series of global mean air temperatures [10, 11, 17–19]. Barreiro et al. analyzed the monthly air temperature anomaly time series via ordinal pattern analysis and also detected LTC over EPWP [62]. Recently, Vera-Valdés investigated LTC in global monthly temperature anomalies by analyzing the autocovariance function and spectral density [63] and found that the degree of LTC were influenced by the long term dynamics of temperature anomalies in the Tropics. In this study, the imprint of ENSO on LTC of SAT anomaly time series is also very obvious [6, 64–66]. Higher values of *α* are distributed in tropical oceans and their extending areas, and this phenomenon could be explained by the higher heat content in the tropical oceans [1]. This finding is also consistent with the distribution of LTC in sea surface temperatures (SST) [20, 67]. Moreover, the differences of *α* values between lands and oceans are obvious (Fig 6). The spatial patterns of STC and LTC are not strictly consistent, i.e. in the polar regions short term persistence exists but there is no evidence of long term persistence in SAT anomaly time series. The results of LTC obtained in this study are basically consistent with those obtained in the above-mentioned literatures. Besides ENSO, it was found that other climate modes such as Pacific Decadal Oscillation (PDO) and Atlantic Multidecadal Oscillation (AMO) could modulate the spatial patterns of LTC in SST time series [68, 69]; however, the imprints of these two climate modes on SAT anomaly time series are not obvious in this study. This discrepancy between SAT and SST deserves a deeper study in future.

NC in an alternative measure for time series nonlinearity, and it has been verified that Δ*σ* is an efficient measure compared to the traditional measure, Δ*α*_*q*_, the width of multifractal spectrum in MFDFA [21]. When MFDFA is applied for thousands of time series, it is not easy to determine a proper range of the scale *n* and order *q* [32, 70]; therefore, the multifractal spectrum for many time series could not be properly obtained [25]. However, the calculation of Δ*σ* does not rely on any regression operation so that this new TP is applicable to all time series. In addition, the applications of MFDFA should consider the finite-size effect and the PDF effect of *X*_*t*_ [25, 71]; however, the calculation of Δ*σ* is not significantly affected by the finite size and the TPs of HVG mapped complex networks are not dependent on PDF of *X*_*t*_ [21]. In this study, NC is considered as the proxy of multifractality [21]. Multifractal analysis has been conducted for air, land, and sea temperature series, where multifractality had been detected using the classical MFDFA method [72–75]. Specifically, the higher degree of NC over the higher latitude regions for SAT time series are consistent with that of higher multifractality for SST time series in [74]. We also found that the higher values of NC were also located near the 15° latitude in the two hemispheres. This phenomenon can be partly explained from the viewpoint of Hadley circulation, also known as the trade wind circulation or planetary Hadley cell. Hadley circulation, a thermally driven circulation system, is prevalent in the Earth’s tropics, specifically spanning approximately 0° to approximately 30° latitude in both hemispheres [76]. It facilitates the development of temperature gradients across various latitudes by transporting warm air from the equator towards the subtropics. The seasonal variations in Hadley circulation are characterized by changes in its intensity and position, primarily influenced by the seasonal distribution of solar radiation and corresponding alterations in surface temperature [77].

Climate time series prediction is a critical task for understanding and forecasting the patterns and trends of complex climate system. Relative to seasonal variations of air temperature, SAT anomalies are of greater concern from a long-term forecasting perspective [78]. By analyzing the serial correlations in global SAT time series, we can effectively improve the ability of climate change prediction since serial correlations are non-negligible for most climate variables [17, 79]. Mei et al. [80] showed that the changing LTC as an early warning indicator of abrupt climate change resulting from a critical transition. Under the background of global climate change, machine learning and other artificial intelligence methods have been widely applied in climate prediction [81, 82]. Artificial Neural Networks (ANN) and Deep Learning (DL) are two representative AI methods in climate predictions [83, 84]. As an extension of recurrent neural networks (RNNs), Long Short Term Memory (LSTM) networks were designed to model chronological sequences and their long-range dependencies more precisely than conventional RNNs. LSTM also performed well in climate time series prediction [85]. We speculate that the three kinds of serial correlations detected in this study should be properly considered from the two perspectives of climate model evaluation and climate predictions.

## Conclusion

In this study, we first identified the STC in surface air temperature anomaly time series, reflecting the impacts of synoptic-scale weather systems. To achieve this, we employed the first-order autoregression method and estimated the persistence length based on ACFs. On this temporal scale, we confirmed the effects of atmospheric Rossby waves in extratropical regions and the EPWP. Next, we detected LTC using the conventional DFA method. We also verified the effects of long-term climate variability, such as the ENSO, on LTC, particularly over the Eastern Pacific. The difference in land-atmosphere and ocean-atmosphere interactions emerged as another factor contributing to the global pattern of LTC in SAT anomalies. Finally, we applied the HVG algorithm to map the SAT anomaly time series onto complex networks. In contrast to previous studies, we found no definitive relationship between the topological parameter, specifically the Spearman correlation between the original time series and degree series, and LTC in global SAT anomaly time series. However, we calculated another TP, (Δ*σ*), to quantify nonlinear correlation in the global SAT anomaly time series. The universality of nonlinear correlation was validated, with higher degrees of NC observed over higher latitude regions and subtropical regions influenced by Hadley circulation.

The uncertainty surrounding the primary findings of this study stems from two primary factors. Firstly, the use of reanalysis datasets introduces a level of uncertainty, as the short-term variability may not be accurately captured by the SAT time series. However, we postulate that this uncertainty does not materially impact the results related to STC and LTC. Secondly, seasonal variation resulting from planetary revolution may contribute to uncertainty. Despite the removal of annual cycles from the original time series, the AR(1) and ACFs results could still potentially be influenced by seasonal effects. These uncertainties warrant further exploration in future research. In summary, our analysis detected serial correlations across multiple scales in global SAT time series, and these correlations are associated with distinct climatological processes and mechanisms.

## Supporting information

S1 File The illustrative examples of computer codes and related data. The computer codes and associated data to illustrate the HVG analysis and Monte Carlo test of the topological parameter the HVG mapped complex network.
(ZIP)

S1 Appendix List of abbreviations in this study. (PDF)
