# Supplementary material for: Multiple serial correlations in global air temperature anomaly time series
Source: PLoS One. 2024 Jul 9;19(7):e0306694. doi: 10.1371/journal.pone.0306694 (PMC11232996; doi:10.1371/journal.pone.0306694)
Supplement: S1 Appendix — (PDF) [file pone.0306694.s002.pdf]

S1 Appendix. List of abbreviations in this study.

Table 1. List of abbreviations.

Abbreviation	Definition
SAT	Surface air temperature
STC	Short term correlation
LTC	Long term correlation
NC	Nonlinear correlation
PDF	Probability distribution function
$AR(1)$	First-order autoregression
VG	Visibility graph
HVG	Horizontal visibility graph
ACF	Autocorrelation function
TP	Topological parameter
DFA	Detrended fluctuation analysis
MFDFA	Multifractal detrended fluctuation analysis
fGN	Fractional Gaussian noise
fBm	Fractional Brownian motion
ENSO	El Niño–Southern Oscillation
EPWP	Eastern Pacific Warm Pool
SST	Sea surface temperature
PDO	Pacific Decadal Oscillation
AMO	Atlantic Multidecadal Oscillation
ANN	Artificial neural networks
RNNs	recurrent neural networks
LSTM	Long Short Term Memory
